# Comment on ‘Factors Associated With Skeletal Muscle Mass in Middle‐Aged Men Living With HIV’ by Xu et al.

**DOI:** 10.1002/jcsm.13655

**Published:** 2024-11-06

**Authors:** Wanfeng Qian, Xiaodong Zhou

**Affiliations:** ^1^ Department of Orthopedics The Affiliated Hospital of Shao Xing University Shaoxing China

**Keywords:** factors, HIV, skeletal muscle mass

Dear Editor,

We have read a recent article [1] in *J Cachexia Sarcopenia Muscle* with great interest. This study aimed to determine the prevalence of low muscle mass within people living with HIV (PLWH) and to identify associated factors. By using multivariate logistic regression analysis, they identified antiretroviral medication types, specifically Zidovudine; BMI and NRI can be independent risk factors for low muscle mass in men with HIV. Despite these definite results, we would like to point out some statistical concerns in this study while the predictors of their research may not be accurate.

First, before the commentary, we want to reiterate the fundamental statistical rule; there should be 10 events (outcome of interest) for 1 variable to be tested for the predictor or risk factor logistic regression analysis [2, 3, 4, 5]. Thus, 27 low muscle mass cases at most analysed three variables in this study. Surprisingly, there were 12 variables in Table 3 (quintile group) of this study when analysed the factors associated with risk of low muscle mass estimated by multivariate logistic regression analysis. Thus, a fourfold overfitted multivariable analysis could not obtain accurate factor results in this study; otherwise, 120 (12 × 10) low muscle mass cases are needed for the factors' statistical analysis. Additionally, a similar overfitted analysis was also found in the AWGS criteria group in Table 3 (27 low muscle mass cases were used to analyse 8 variables).

Second, we are curious about why not the author used the univariate logistic regression analysis to reduce the factors before the final multivariate logistic regression analysis in Table 3. It could obtain more reliable results.

Third, we are curious about the rationale for selecting these 8 variables (quintile group) or 12 variables (AWGS criteria) for the predictor analysis in Table 3, were they chosen at random or according to their clinical experience? According to the commonly accepted statistical rule, the author could use the significant variables in Table 2 (statistical analyses between the normal muscle and low muscle based on quintile group or AWGS criteria) for the factors analysis in Table 3. But the authors seem not to use these significant variables in Table 2 for the factors analysis in Table 3. Thus, selecting the variables at random could not obtain an accurate risk factor in this study.

Lastly, it is a great honour to comment on Xu et al.'s despite these comments.

## Ethics Statement

“The authors have nothing to report.

## Consent

All authors reviewed this manuscript and agreed to submit this manuscript.

## Conflicts of Interest

The authors declare no conflicts of interest.

## References

[1] Y. Xu , D. Wang , P. Chen , et al., “Factors Associated With Skeletal Muscle Mass in Middle‐Aged Men Living With HIV,” Journal of Cachexia, Sarcopenia and Muscle 15 (2024): 1965–1975, 10.1002/jcsm.13545. 39015948 PMC11446698

[2] A. Pate , R. D. Riley , G. S. Collins , et al., “Minimum Sample Size for Developing a Multivariable Prediction Model Using Multinomial Logistic Regression,” Statistical Methods in Medical Research 32 (2023): 555–571, 10.1177/09622802231151220. 36660777 PMC10012398

[3] M. van Smeden , K. G. Moons , J. A. de Groot , et al., “Sample Size for Binary Logistic Prediction Models: Beyond Events per Variable Criteria,” Statistical Methods in Medical Research 28 (2019): 2455–2474, 10.1177/0962280218784726. 29966490 PMC6710621

[4] R. D. Riley , J. Ensor , K. I. E. Snell , et al., “Calculating the Sample Size Required for Developing a Clinical Prediction Model,” British Medical Journal (Clinical Research Edition) 368 (2020): m441, 10.1136/bmj.m441. 32188600

[5] R. D. Riley , K. I. Snell , J. Ensor , et al., “Minimum Sample Size for Developing a Multivariable Prediction Model: PART II ‐ Binary and Time‐To‐Event Outcomes,” Statistics in Medicine 38 (2019): 1276–1296, 10.1002/sim.7992. 30357870 PMC6519266

